# Deoxyribonucleotide Triphosphate Metabolism in Cancer and Metabolic Disease

**DOI:** 10.3389/fendo.2018.00177

**Published:** 2018-04-18

**Authors:** Raquel Buj, Katherine M. Aird

**Affiliations:** Department of Cellular and Molecular Physiology, Penn State College of Medicine, Hershey, PA, United States

**Keywords:** purines, pyrimidines, c-Myc, p53, mTORC1, diabetes, obesity

## Abstract

The maintenance of a healthy deoxyribonucleotide triphosphate (dNTP) pool is critical for the proper replication and repair of both nuclear and mitochondrial DNA. Temporal, spatial, and ratio imbalances of the four dNTPs have been shown to have a mutagenic and cytotoxic effect. It is, therefore, essential for cell homeostasis to maintain the balance between the processes of dNTP biosynthesis and degradation. Multiple oncogenic signaling pathways, such as c-Myc, p53, and mTORC1 feed into dNTP metabolism, and there is a clear role for dNTP imbalances in cancer initiation and progression. Additionally, multiple chemotherapeutics target these pathways to inhibit nucleotide synthesis. Less is understood about the role for dNTP levels in metabolic disorders and syndromes and whether alterations in dNTP levels change cancer incidence in these patients. For instance, while deficiencies in some metabolic pathways known to play a role in nucleotide synthesis are pro-tumorigenic (e.g., p53 mutations), others confer an advantage against the onset of cancer (G6PD). More recent evidence indicates that there are changes in nucleotide metabolism in diabetes, obesity, and insulin resistance; however, whether these changes play a mechanistic role is unclear. In this review, we will address the complex network of metabolic pathways, whereby cells can fuel dNTP biosynthesis and catabolism in cancer, and we will discuss the potential role for this pathway in metabolic disease.

## Introduction

The maintenance of deoxyribonucleotide triphosphate (dNTP) pools is critical for multiple cellular pathways. For instance, imbalances in dNTPs are associated with genomic instability (1). Likewise, they have also been shown to disturb mitochondrial DNA (mtDNA) and consequently mitochondrial fitness, which may lead to mitochondrial diseases (MDs), such as diabetes, obesity, and cancer (2). Additionally, disorders of purine and pyrimidine metabolism (DPPM) profoundly affect cell metabolism, underlying the importance of nucleotides for cell behavior (3). Thus, both nucleotide synthesis and degradation must be exquisitely fine-tuned. In this review, we will focus on synthesis of dNTPs and the consequences of dNTP pool imbalances in cancer and MDs.

## Healthy dNTP Pools

A correct balance of dNTPs is necessary for the prevention of multiple pathologies. A healthy cell must maintain two asymmetric and spatial-temporal dNTP pools; one for nuclear DNA synthesis and repair and another for mtDNA replication and repair. Disruptions in dNTP balance are associated with enhanced mutagenesis, leading to genomic instability, which promotes cancer (4), and may have a role in metabolic disease (5).

Cytosolic dNTP pool concentrations positively correlate with the cell cycle. In fact, the amount of dNTPs at the beginning of S-phase is not enough for a complete DNA duplication (6). The S-phase increase in dNTPs is necessary for faithful nuclear DNA replication. mtDNA is replicated continuously in post-mitotic cells, and faithful maintenance of mtDNA also depends on correctly balanced dNTPs (7). Thus, both proliferating and non-proliferating cells need to fine-tune nucleotide and dNTP synthesis to allow for both nuclear and mtDNA replication and repair to maintain the health of the cell.

### Anabolism and Catabolism of Nucleotides

Cells possess two biosynthetic pathways to produce dNTPs: *de novo* and salvage (8). Purines and pyrimidines arise from two different *de novo* pathways that generate nucleotides starting from raw material (glucose, glutamine, aspartate, and HCO_3_) (9). The *de novo* nucleotide synthesis pathway is highly energy-intensive (9). Therefore, cells have developed a more energy-efficient route to synthesize nucleotides, termed the salvage pathway (10). The salvage pathway acts as a recycling plant taking free nitrogen bases and nucleosides arising from nucleic acid breakdown and diet (9). Nucleosides are hydrophilic compounds, thus proper function of nucleoside transporters (SLC29 and SLC28 families) is an essential requirement for salvage pathway function (11). Ribonucleotides obtained by either pathway can be reduced to their deoxyribonucleotide counterpart in a reaction catalyzed by ribonucleotide reductase (RNR) (12).

**Figure 1:**
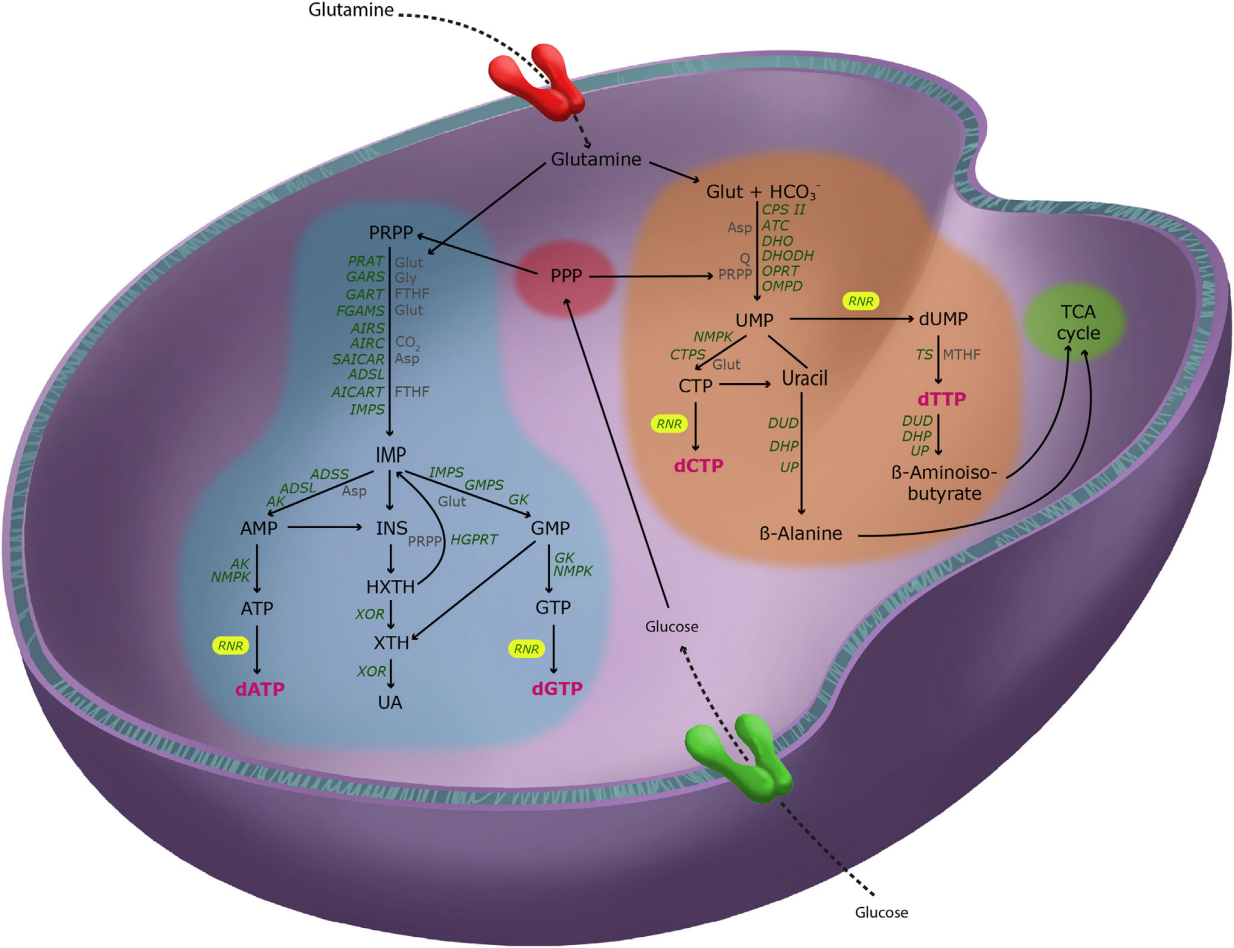
Pathways of deoxyribonucleotide metabolism in mammalian cells. Simplified representation of purine (blue) and pyrimidine (orange) metabolism and their crosstalk with the major metabolic pathways, the pentose phosphate pathway [(PPP), red] and the tricarboxylic acid cycle (green). Key metabolic enzymes (green), their principal reactive substrates (gray), and the four deoxyribonucleotide triphosphate (dNTP) end-products (magenta) are shown. Glucose and glutamine feed into both purine and pyrimidine metabolism to donate carbons and nitrogens to all dNTPs. Abbreviations: RAT, phosphoribosylpyrophosphate amidotransferase; GARS, glycinamide ribonucleotide synthetase; GART, glycinamide ribonucleotide transformylase; FGAMS, phosphoribosylformyl-glycineamide synthetase; AIRS, phosphoribosylaminoimidazole synthetase; AIRC, phosphoribosylaminoimidazole carboxylase; SAICAR, phosphoribosylaminoimidazole-succinocarboxamide; ADSL, adenylosuccinate lyase; AICART, phosphoribosylaminoimidazolecarboxamide formyltransferase; IMPS, inosine monophosphate synthase; ADSS, adenylosuccinate synthetase; AK, adenylate kinase; NMPK, nucleotide monophosphate kinase; IMPS, inosine monophosphate dehydrogenase; GMPS, guanosine monophosphate synthetase; GK, guanylate kinase; XOR, xanthine oxidoreductase; HGPRT, hypoxanthine-guanine phosphoribosyltransferase; RNR, ribonucleotide reductase; CPS II, carbamoyl phosphate synthetase II; ATC, aspartate carbamoyltransferase; DHO, dihydroorotase; DHOD, dihydroorotase dehydrogenase; OPRT, orotate phosphoribosyltransferase; OMPD, orotidine monophosphate decarboxylase; CPTS, cytidine triphosphate synthetase; TS, thymidylate synthase; DUD, dihydrouracil dehydrogenase; DHP, dihydropyrimidinase; UP, ureidopropionase; Glut, glutamine; Gly, glycine; FTHF, *N*10-formyltetrahydrofolate; Asp, aspartate; PRPP, phosphoribosylpyrophosphate; Q, ubiquinone; MTHF, *N*5,*N*10-methylenetetrahydrofolate.

Turnover of RNA and other nucleotides occurs regularly to maintain homeostasis. Human cells cannot break down the purine ring. Purine catabolism involves a sequence of three reactions in which nucleotides are stripped step-by-step from their phosphates and sugar to finally become oxidized to the end product uric acid (UA), which is excreted into the urine (13). Conversely, uracil and thymidine rings can be completely degraded to β-alanine and β-aminoisobutyrate, respectively. Subsequently, both metabolites can be excreted or transformed into intermediates of the tricarboxylic acid (TCA) cycle (14). Biosynthesis and catabolism of nucleotides and dNTPs are highlighted in Figure 1.

## Impaired Nucleotide Metabolism in Cancer and Metabolic Disease

Deregulation of nucleotide metabolism is associated with a broad spectrum of pathological conditions, including cancer and MDs (15–17). Virtually all metabolic pathways have been implicated in dNTP biosynthesis. Thus, *de novo* and salvage pathways, as well as all involved anapleurotic reactions (Figure 1), need to be highly cross-regulated.

It is well known that cancer cells must increase dNTP biosynthesis (18) to ensure rapid replication of the genome (17). This occurs through a variety of pathways (discussed below). In contrast, MDs are caused by congenital or acquired genetic defects in metabolic enzymes. DPPM are due to abnormalities in the biosynthesis, interconversion, and degradation of nucleotides (19). DPPM have a wide variety of clinical presentations, highlighting the importance of proper nucleotide metabolism for cell and organism function (15). Alterations in nucleotide metabolism are also present in other metabolic-related pathological conditions, such as diabetes, obesity, and insulin resistance (20–22) (Table 1). In this section, we will summarize some important features affecting nucleotide metabolism in cancer and MDs.

**Table 1:** Genes, protein families, and pathways discussed in this review: role in deoxyribonucleotide triphosphate (dNTP) metabolism and expression in cancer and metabolic disease.

Gene/family/pathway	Known role in dNTP metabolism	Expression in cancer	Expression in metabolic disease
Purine/pyrimidine synthesis pathway	Necessary for *de novo* dNTP biosynthesis (8)^a^	Increased (23) or mutated (24, 25)	Heptatic steatosis (uridine metabolism) (↓) (26)Diabetes^b^ (↓) (27)
*MTOR*	Promotes glucose uptake (28); promotes *de novo* nucleotide biosynthesis (29, 30)	Increased (31)	Diabetes (↑) (32)Obesity (↑) (33)
*MYC*	Induces glucose uptake and utilization (34); transcriptionally regulates nucleotide metabolic enzymes (23, 35)	Increased (oncogene) (23)	Insulin resistance (↑)^c^Obesity (↑)^c^ (36)
*TP53*	Negative regulator of pentose phosphate pathway through G6PD (37); gain-of-function mutations increase gene transcription of genes for dNTP synthesis (38)	Decreased or mutated (tumor suppressor) (39)	Insulin resistance (↑)Glucose intolerance (mut) (5)Mitochondrial changes (mut)^d^ (40)
PI3K-AKT pathway	Oncogenic activation promotes glucose and glutamine uptake and catabolism (41)	Increased (oncogenes) (41)	Diabetes (↑) (42)Nonalcoholic fatty liver disease (↑) (43)Obesity (↑) (44)
ERK-MAPK pathway	Regulation of CPS II in *de novo* pyrimidine synthesis (45)	Increased (oncogenes) (46)	Diabetes (↑) (47)Obesity (↑) (48)
*G6PD*	Rate-limiting for ribose-5-phosphate synthesis from the PPP (49)^a^	Increased or mutated (50)	Obesity (↑) (51)Diabetes (↑) (52)
*RRM1*	Catalytic subunit of the ribonucleotide reductase (RNR); catalyzes the reduction of deoxyribonucleotides from ribonucleotides (12)^a^	Increased or decreased (53)	Unknown
*RRM2*	Regulatory subunit of RNR (12); S-phase regulated (54); rate-limiting enzyme in the reduction of deoxyribonucleotides from ribonucleotides (55)^a^	Increased (oncogene) (53)	Unknown
*RRM2B*	Regulatory subunit of the RNR (56); formation of deoxyribonucleotides from ribonucleotides for DNA damage repair and mitochondrial DNA (mtDNA) replication (57–59)^a^	Increased or decreased (53)	Mitochondrial disorders (↓) (60)
SLC25 family	Mitochondrial nucleoside transporters (61)Important for mtDNA pools through the salvage pathway (62)	Increased (63)	Mitochondrial disease (mut)^e^Mitochondrial dysfunction (↓)^f^ (61)
SLC29 and SLC28 families	Nucleoside transporters that are important for the salvage pathway (11, 64, 65)	Increased (11)	Diabetes (mut)^g^ (66)
*TK2*	Phosphorylates deoxycytidine to generate dCTP (67)	Unknown	Mitochondrial disease (↓) (68)
*DGUOK*	Catalyzes the conversion of deoxyguanosine to dGMP (67)^a^	Mutated^h^ (69, 70)	Mitochondrial disease (mut) (69)
*TWNK*	Mitochondrial helicase (71)	Unknown	Mitochondrial dysfunction (mut) (72, 73)
*POLG*	Catalytic subunit of the mitochondrial DNA polymerase (74)	Mutated (75–77)	Mitochondrial disease (mut) (77–79)
*Ataxia-telangiectasia mutated*	Increases glucose/glutamine uptake and inhibits the PPP (80)	Mutated (81)	Mitochondrial dysfunction (mut)^i^Insulin resistance (mut)^i^ (82)
*XOR*	Catalyzes the conversion of xanthine to uric acid (83)^a^	Increased^j^ or decreased (84)	Metabolic syndrome (mut)^k^Insulin resistance (mut)^k^Diabetes (mut)^k^Fatty liver disease (mut)^k^ (85)

*^a^These genes/pathways are shown in Figure 1*.

*^b^These studies show that purines and pyrimidines are downregulated in diabetes. It is not known whether changes in purine or pyrimidine synthesis genes are the mechanism behind this observation*.

*^c^Increased MYC expression counteracts insulin resistance and obesity*.

*^d^Occurs in patients with Li–Fraumeni syndrome*.

*^e^*SLC25A4* (86)*.

*^f^*SLC25A33* and *SLC25A36* have only been tested in mouse models (87, 88)*.

*^g^SLC29A3 is the only gene in this family that has been found to affect metabolic disease*.

*^h^While the data are limited, some patients with DGUOK mutations have hepatocellular carcinoma*.

*^i^Occurs in patients with ataxia-telangiectasia*.

*^j^Increased XOR expression/activity is likely important for cancer initiation; however, XOR expression is decreased in most established tumors*.

*^k^Occurs in patients with XOR deficiency*.

### Deregulation of Major Growth Signaling Pathways Leads to Nucleotide Pool Imbalances in Cancer and Metabolic Disease

The main growth signaling pathways (PI3K-AKT and ERK1/2-MAPK) are induced and maintained during metabolic reprogramming of cancer (18). Additionally, deregulation of these pathways may contribute to different MDs, including diabetes, obesity, or steatosis resistance (33, 89, 90). These pathways sense and orchestrate nutrient utilization; therefore, is not surprising that alterations in these pathways affect energy and biomass production and cause a broad variety of diseases.

mTOR is a central signaling pathway that integrates environmental inputs (e.g., nutrients and hormones) into downstream pathways to control many cellular processes (91). This includes regulation of metabolism, growth, and survival (32). Indeed, the mTORC1/2 pathway not only promotes glucose uptake and protein and lipid biosynthesis, but also promotes nucleotide biosynthesis (29, 30) and uptake of nucleosides through transporters (88). At least one member of this pathway is altered in 38% of human cancer (92). Altered metabolism induced by aberrant mTORC1 activation has also been shown to play a role in diabetes and obesity (32, 93).

c-Myc, one of the most commonly altered proteins in human cancer, is also regulated by PI3K-AKT and ERK1/2-MAPK pathways (94). c-Myc is a highly pleiotropic transcription factor considered a master regulator of cell metabolism (34, 35) through regulation of glycolysis, glutamine metabolism, and mitochondrial biogenesis (95, 96). Indeed, c-Myc has been shown to induce hepatic glucose uptake and utilization, while blocking gluconeogenesis and ketogenesis, suggesting a counteracting effect of c-Myc in obesity and insulin resistance (36, 97). In addition to regulating glucose and glutamine, substrates for purine and pyrimidine biosynthesis (Figure 1) (98), c-Myc also transcriptionally regulates nucleotide metabolic enzyme gene expression (35). Thus, deregulation in c-Myc acutely alters nucleotide homeostasis in cancer (99), and it is interesting to speculate that the role of c-Myc in MDs is also related to nucleotide metabolism.

Previous publications from our laboratory and others have shown that DNA damage and DNA damage response (DDR) proteins regulate dNTP biosynthesis in the context of cancer (80, 100, 101). Interestingly, upregulation of p53, a key player in the DDR, in adipose tissue is associated with increased inflammation and insulin resistance (102). Notably, wild-type p53 negatively regulates G6PD activity (37), the rate-limiting enzyme of the pentose phosphate pathway and one of the most important sources of nucleotides (103). Upregulation of G6PD correlates with functional defects in liver, heart, and pancreas of obese and diabetic animals (104). Although the relationship between G6PD upregulation and increased oxidative stress has been studied in MD (105), the implication for nucleotide metabolism has not yet been addressed. More research is needed to understand the contribution of dNTP imbalances due to G6PD deregulation in diabetes and obesity.

An imbalance in nucleotides has been shown in two different studies related to diabetes (106, 107). Additionally, pyrimidine metabolism has been linked to fatty liver (26). Interestingly, increasing evidence suggests a link between obesity, a risk factor for non-alcoholic fatty liver disease (108), and cancer. Obese patients show many cancer-promoting features, such as chronic low-level inflammation (109), insulin-resistance/diabetes (110), and deregulation of mTORC1 (111). Although the contribution of deregulated nucleotide pools promoting cancer has been extensively demonstrated (18, 112–115), their role in MD and metabolic-related diseases has not yet been elucidated. Based on these recent studies, we speculate that deregulation of nucleotide pools may in part contribute to the altered metabolic landscape promoting obesity and diabetes. Studying the implications of altered nucleotide pools in these diseases would open a therapeutic window based on modulation of nucleotide metabolism.

### RNR in Cancer and Metabolic Disease

Ribonucleotide reductase reduces ribonucleotides to the corresponding deoxyribonucleotides (116, 117). In mammals, RNR is a tetrameric enzyme composed of two homodimeric subunits, RRM1 and RRM2. Whereas, RRM1 is continuously expressed throughout the cell-cycle, expression of RRM2 is activated upon entry into S-phase (54, 118). Additionally, RRM2 is rapidly degraded *via* the proteasome in G2 (12, 119). Thus, RRM2 is considered rate-limiting for RNR activity. RRM2B (RNR subunit M2B) is an alternative M2 subunit that is induced by p53 activation in response to DNA damage (56). RRM2B is not cell-cycle regulated *per se*, but it plays key roles in enhancing dNTP synthesis in cells under stress (120–122) and mediating mtDNA synthesis and repair (57–59).

The role of RNR in cancer is clear as it was one of the first identified DNA damage-induced enzymes (123). While RRM2 overexpression is tumorigenic, leading to lung neoplasms *in vivo*, RRM1 reduces tumor formation, migration, and metastasis [reviewed in Ref. (53)]. Previous studies from our lab and others have shown the potential of RRM2 as a prognostic and diagnostic biomarker in multiple cancers (112, 124–127). However, the utility of RRM1 and RRM2B as a tumor biomarker is still unclear [reviewed in Ref. (53)].

Although there is no study directly linking RNR with MD, RRM2B is required for mtDNA synthesis and healthy mitochondrial function (57). Deregulated mitochondria are associated with a higher risk of diabetes and obesity (discussed below). Therefore, it is possible that RNR function is linked to these MDs (Table 1). More mechanistic studies will be needed to determine the role for RNR in obesity and diabetes.

### Mitochondrial Dysfunction in dNTP Pool Disruption During Cancer and Metabolic Disease

The mitochondria are one of the most important organelles for eukaryotic function (128). In addition to the production of ATP through oxidative phosphorylation, mitochondria are also the scaffold of several metabolic reactions for cellular building block synthesis (e.g., fatty acid beta-oxidation, one-carbon/folate cycle, TCA cycle, amino acid metabolism, etc.) (129). Hence, altered mitochondrial behavior has a broad impact on cellular metabolism.

Maintenance of mitochondrial dNTP pools is critical for proper mtDNA function. Alterations in nuclear genes involved in transport of cytosolic dNTPs (e.g., SLC25A4), the salvage nucleotide biosynthesis in the inner mitochondrial membrane (e.g., TK2 and DGUOK), and genes involved in mtDNA replication (e.g., TWNK and POLG) are implicated in both cancer and metabolic syndromes (63, 68, 77–79, 130–133). Moreover, dysfunction in the electron transport chain induces oxidative stress, which has been associated with impaired one-carbon metabolism (134, 135), an essential anapleurotic pathway for both purine and pyrimidine nucleotides. Mitochondrial genomic instability due to increased levels of reactive oxygen species (ROS) and/or mutations in mtDNA or nuclear genes involved in mitochondria function are underlying factors of MDs, and contribute to cancer and diabetes (136). Alterations in genes discussed above that are important for dNTP homeostasis and mitochondrial function are highlighted in Table 1.

Although the link between mitochondrial dysfunction and MD has been studied for the past two decades, the results are contradictory (137). These contradictory results mainly arise from the complex relationship between mitochondria and metabolism, but also from the lack of global and standardized methodological strategies to phenotype insulin-resistance in humans (138). Dysregulation of nucleotide metabolism is an important aspect of mitochondrial dysfunction; therefore, their role in MDs should not be ignored.

### Relationship Between DPPM and Cancer

It is clear that cancer is a metabolic disease; however, a predisposition to cancer is not a foregone conclusion in patients with DPPM, who by definition have alterations in nucleotide supplies. Interestingly, while deficiencies in some metabolic pathways known to play a role in nucleotide synthesis are pro-tumorigenic, others confer an advantage against the onset of cancer. This highlights the large variability in the clinical presentation of these disorders.

Alterations in p53 or ataxia-telangiectasia mutated (ATM) lead to metabolic changes and predispose patients to cancer. Patients with germline *TP53* (encoding for p53) mutations have Li–Fraumeni syndrome and are predisposed to cancer (139, 140). Interestingly, a recent report showed that nucleotide metabolism is regulated by the gain-of-function activity of mutant p53 (38). Consistently, wild-type p53 negatively regulates G6PD and PPP activity to decrease dNTP synthesis (37). Similarly, our group has previously shown that ATM (mutated in some ataxia-telangiectasia patients) inactivation increases glucose uptake and enhances glucose flux through the PPP and ultimately increases dNTP biosynthesis (Figure 1) (80, 141). Indeed, patients with ATM mutations show alterations in glucose homeostasis (142, 143). It is well-known that these patients have an increased susceptibility to cancer (144). It is interesting to speculate that alterations in dNTP metabolism may play a role in the cancer predisposition in these patients; however, further studies are needed to support this notion.

Other DPPM confer a tumor suppressive benefit. For instance, patients with G6PD deficiency have a reduced risk of some cancers (145–147) (Table 1). This suggests that hyperactivity of dNTP synthesis is more likely to increase cancer risk than deficiencies in synthesis.

Finally, some DPPM have both a pro- and anti-tumorigenic effect. Deficiency in xanthine oxidoreductase (XOR), the enzyme that catalyzes the last step in purine catabolism (Figure 1), increases UA (148). There is a dual role for UA in cancer, the so-called the oxidant–antioxidant UA paradox (149). On one hand, extracellular UA is a potent ROS scavenger, thus protecting cells against oxidative stress (150). On the other hand, high intracellular levels of UA in a XOR-deficient cellular background promote dNTP biosynthesis and tumor growth by shuttling XOR precursors (xanthine and hypoxanthine) into the purine salvage pathway (149). Additionally, intracellular UA is pro-inflammatory by inducing NADPH-oxidases that lead to oxidative stress and cancer (151, 152). This again emphasizes the complex nature of these disorders in relation to cancer (Table 1).

Together, the lack of consensus in predisposition to cancer in DPPM patients points to the significant redundancy in the dNTP biosynthetic pathways. This should not be surprising due to the fact that dNTP synthesis is critical for organismal survival and, therefore, we have evolved to have multiple metabolic arms feeding into the same pathway. Understanding whether these patients are predisposed or not to cancer will be incredibly important for the clinical management of these patients.

## Therapeutic Modulation of Deoxyribonucleotide Metabolism in Cancer and Metabolic Disease

As described in this review, the balance of dNTPs must be tightly regulated in the cell. Many cancer types show alterations in dNTP levels, supporting their rapid proliferation. Likewise, defective mutations in anabolic and catabolic nucleotide enzymes, causing imbalances in the dNTP pools or in their precursors, are associated with different grades of disease severity in DPPM. Thus, it is not surprising that therapies for both cancer and DPPM focus on restoration of the normal balance of intracellular nucleotides.

Some of the first chemotherapeutic agents were cytotoxic nucleoside analogs and nucleobases (e.g., thiopurines and fluoropyrimidines) (153). These antimetabolites have a similar molecular structure to endogenous nucleotides and interfere with nucleotide metabolic pathways and DNA/RNA synthesis (154). Inhibitors of RNR were one of the first cancer therapies [reviewed in Ref. (53)] and are still used today. For instance, gemcitabine, a chemotherapeutic nucleoside analog, is used in pancreatic adenocarcinoma, but also in breast, bladder, and non-small cell lung cancer (155). Unfortunately, resistance to gemcitabine in common, often through an increase in nucleotide synthesis pathways or transport of nucleosides (156). Other successful chemotherapeutic regimens include methotrexate, which reduces substrates for purine and pyrimidine biosynthesis (157). Finally, specific inhibition of enzymes in the *de novo* pathway and/or in anapleurotic reactions (glucose and glutamine metabolism) has also been used as adjuvant therapies in cancer (154).

The spectrum of nucleotide therapies for DPPM is much broader in scope due to the high variability of deficiencies (3). Thus, deficiencies resulting in the overproduction of UA are treated with allopurinol, an inhibitor of xanthine oxidase (16). In other cases, patients can be treated with oral supplements of specific nucleotides they are lacking (16). What is clear is that cancer patients with DPPM cannot be treated with antimetabolites such as 5-fluoro-uracil due to severe side effects (19). This suggests that cancer patients, DPPM must remain above a certain threshold of nucleotide pools to remain healthy. Finally, no nucleotide therapies are currently used for MDs, such as diabetes or obesity. More studies will need to be performed to determine whether nucleotide metabolism plays a contributing role to these pathologies before these types of therapies can be tested.

## Conclusion

For decades researchers and clinicians alike have recognized the importance of fine-tuned dNTP levels for cellular homeostasis, as shown by the number of anti-cancer therapies based on the abolishment of nucleotide synthesis. In addition, the broad range of pathologies associated with congenital defects in nucleotide metabolic enzymes further demonstrates the importance of healthy intracellular dNTP levels. However, the association between cancer and MD and whether nucleotide pools are interconnected in these pathologies remains unclear. Future work will need to focus on mechanistic and population-based studies to determine whether nucleotide pool imbalances in MD lead to changes in cancer predisposition and whether targeting these pathways for cancer therapy affects metabolic homeostasis and function in normal cells.

## Author Contributions

RB and KA conceived of and wrote the manuscript.

## Conflict of Interest Statement

The authors declare that the research was conducted in the absence of any commercial or financial relationships that could be construed as a potential conflict of interest.

## References

[1] Zeman MK Cimprich KA Causes and consequences of replication stress. Nat Cell Biol (2014) 16(1):2–9. 10.1038/ncb2897 24366029 PMC4354890

[2] Gilkerson R. Commentary: mitochondrial DNA damage and loss in diabetes. Diabetes Metab Res Rev (2016) 32:672–4. 10.1002/dmrr.2833 27253402 PMC5248653

[3] Van Den Berghe G Vincent MF Marie S Disorders of purine and pyrimidine metabolism. In: Saudubray JM Berghe GD Walter JH, editors. Inborn Metabolic Diseases: Diagnosis and Treatment. Berlin, Heidelberg: Springer-Verlag (2012). p. 499–518.

[4] Mathews CK. DNA precursor metabolism and genomic stability. FASEB J (2006) 20:1300–14. 10.1096/fj.06-5730rev 16816105

[5] Shimizu I Yoshida Y Suda M Minamino T. DNA damage response and metabolic disease. Cell Metab (2014) 20:967–77. 10.1016/j.cmet.2014.10.008 25456739

[6] Kohalmi SE Glattke M McIntosh EM Kunz BA. Mutational specificity of DNA precursor pool imbalances in yeast arising from deoxycytidylate deaminase deficiency or treatment with thymidylate. J Mol Biol (1991) 220:933–46. 10.1016/0022-2836(91)90364-C 1880805

[7] Pai CC Kearsey SE. A critical balance: DNTPs and the maintenance of genome stability. Genes (Basel) (2017) 8(2):E57. 10.3390/genes8020057 28146119 PMC5333046

[8] Blakley RL Vitols E The control of nucleotide biosynthesis. Annu Rev Biochem (1968) 37:201–24. 10.1146/annurev.bi.37.070168.001221 4875716

[9] Lane AN Fan TWM. Regulation of mammalian nucleotide metabolism and biosynthesis. Nucleic Acids Res (2015) 43(4):2466–85. 10.1093/nar/gkv047 25628363 PMC4344498

[10] Moffatt BA Ashihara H Purine and pyrimidine nucleotide synthesis and metabolism. Arab B (2002) 1:e0018 10.1199/tab.0018 PMC3243375 22303196

[11] Young JD Yao SYM Baldwin JM Cass CE Baldwin SA. The human concentrative and equilibrative nucleoside transporter families, SLC28 and SLC29. Mol Aspects Med (2013) 34:529–47. 10.1016/j.mam.2012.05.007 23506887

[12] Fairman JW Wijerathna SR Ahmad MF Xu H Nakano R Jha S Structural basis for allosteric regulation of human ribonucleotide reductase by nucleotide-induced oligomerization. Nat Struct Mol Biol (2011) 18:316–22. 10.1038/nsmb.2007 21336276 PMC3101628

[13] Maiuolo J Oppedisano F Gratteri S Muscoli C Mollace V. Regulation of uric acid metabolism and excretion. Int J Cardiol (2016) 213:8–14. 10.1016/j.ijcard.2015.08.109 26316329

[14] Wasternack C Degradation of pyrimidines and pyrimidine analogs-pathways and mutual influences. Pharmacol Ther (1980) 8:629–51. 10.1016/0163-7258(80)90079-0 6992162

[15] Van Gennip AH Defects in metabolism of purines and pyrimidines. Ned Tijdschr Klin Chem (1999) 24:171–5.

[16] Jurecka A. Inborn errors of purine and pyrimidine metabolism. J Inherit Metab Dis (2009) 32:247–63. 10.1007/s10545-009-1094-z 19291420

[17] Kohnken R Kodigepalli KM Wu L. Regulation of deoxynucleotide metabolism in cancer: novel mechanisms and therapeutic implications. Mol Cancer (2015) 14:176. 10.1186/s12943-015-0446-6 26416562 PMC4587406

[18] Pavlova NN Thompson CB. The emerging hallmarks of cancer metabolism. Cell Metab (2016) 23:27–47. 10.1016/j.cmet.2015.12.006 26771115 PMC4715268

[19] Bakker JA Bierau J Purine and pyrimidine metabolism: still more to learn. Ned Tijdschr voor Klin Chemie en Lab (2012) 37:3–6.

[20] Choi Y-J Yoon Y Lee K-Y Kang Y-P Lim DK Kwon SW Orotic acid induces hypertension associated with impaired endothelial nitric oxide synthesis. Toxicol Sci (2015) 144:307–17. 10.1093/toxsci/kfv003 25601987

[21] Kunjara S Sochor M Ali M Drake A Greenbaum AL McLean P. Pyrimidine nucleotide synthesis in the rat kidney in early diabetes. Biochem Med Metab Biol (1991) 46:215–25. 10.1016/0885-4505(91)90069-W 1723607

[22] Tsushima Y Nishizawa H Tochino Y Nakatsuji H Sekimoto R Nagao H Uric acid secretion from adipose tissue and its increase in obesity. J Biol Chem (2013) 288:27138–49. 10.1074/jbc.M113.485094 23913681 PMC3779712

[23] Satoh K Yachida S Sugimoto M Oshima M Nakagawa T Akamoto S Global metabolic reprogramming of colorectal cancer occurs at adenoma stage and is induced by MYC. Proc Natl Acad Sci U S A (2017) 114:E7697–706. 10.1073/pnas.1710366114 28847964 PMC5604037

[24] Berg M Ågesen TH Thiis-Evensen E Merok MA Teixeira MR Vatn MH Distinct high resolution genome profiles of early onset and late onset colorectal cancer integrated with gene expression data identify candidate susceptibility loci. Mol Cancer (2010) 9:100. 10.1186/1476-4598-9-100 20459617 PMC2885343

[25] Jo YS Oh HR Kim MS Yoo NJ Lee SH. Frameshift mutations of OGDH, PPAT and PCCA genes in gastric and colorectal cancers. Neoplasma (2016) 63:681–6. 10.4149/neo_2016_504 27468871

[26] Le TT Ziemba A Urasaki Y Hayes E Brotman S Pizzorno G. Disruption of uridine homeostasis links liver pyrimidine metabolism to lipid accumulation. J Lipid Res (2013) 54:1044–57. 10.1194/jlr.M034249 23355744 PMC3605981

[27] Pillwein K Reardon MA Jayaram HN Natsumeda Y Elliott WL Faderan MA Insulin regulatory effects on purine- and pyrimidine metabolism in alloxan diabetic rat liver. Padiatr Padol (1988) 23:135–44. 3043317

[28] Cornu M Albert V Hall MN MTOR in aging, metabolism, and cancer. Curr Opin Genet Dev (2013) 23(1):53–62. 10.1016/j.gde.2012.12.005 23317514

[29] Ben-Sahra I Hoxhaj G Ricoult SJH Asara JM Manning BD. mTORC1 induces purine synthesis through control of the mitochondrial tetrahydrofolate cycle. Science (2016) 351:728–33. 10.1126/science.aad0489 26912861 PMC4786372

[30] Ben-Sahra I Howell JJ Asara JM Manning BD. Stimulation of de novo pyrimidine synthesis by growth signaling through mTOR and S6K1. Science (2013) 339:1323–8. 10.1126/science.1228792 23429703 PMC3753690

[31] Guertin DA Sabatini DM Defining the role of mTOR in cancer. Cancer Cell (2007) 12(1):9–22. 10.1016/j.ccr.2007.05.008 17613433

[32] Ardestani A Lupse B Kido Y Leibowitz G Maedler K mTORC1 signaling: a double-edged sword in diabetic β cells. Cell Metab (2018) 27:314–31. 10.1016/j.cmet.2017.11.004 29275961

[33] Khamzina L Veilleux A Bergeron S Marette A. Increased activation of the mammalian target of rapamycin pathway in liver and skeletal muscle of obese rats: possible involvement in obesity-linked insulin resistance. Endocrinology (2005) 146:1473–81. 10.1210/en.2004-0921 15604215

[34] Dang CV MYC on the path to cancer. Cell (2012) 149(1):22–35. 10.1016/j.cell.2012.03.003 22464321 PMC3345192

[35] Liu YC Li F Handler J Huang CR Xiang Y Neretti N Global regulation of nucleotide biosynthetic genes by c-Myc. PLoS One (2008) 3(7):e2722. 10.1371/journal.pone.0002722 18628958 PMC2444028

[36] Riu E Ferre T Hidalgo A Mas A Franckhauser S Otaegui P Overexpression of c-myc in the liver prevents obesity and insulin resistance. FASEB J (2003) 17:1715–7. 10.1096/fj.02-1163fje 12958186

[37] Jiang P Du W Wang X Mancuso A Gao X Wu M P53 regulates biosynthesis through direct inactivation of glucose-6-phosphate dehydrogenase. Nat Cell Biol (2011) 13:310–6. 10.1038/ncb2172 21336310 PMC3110666

[38] Kollareddy M Dimitrova E Vallabhaneni KC Chan A Le T Chauhan KM Regulation of nucleotide metabolism by mutant p53 contributes to its gain-of-function activities. Nat Commun (2015) 6:7389. 10.1038/ncomms8389 26067754 PMC4467467

[39] Liu J Zhang C Feng Z. Tumor suppressor p53 and its gain-of-function mutants in cancer. Acta Biochim Biophys Sin (Shanghai) (2014) 46(3):170–9. 10.1093/abbs/gmt144 24374774 PMC3932832

[40] Wang P-Y Ma W Park J-Y Celi FS Arena R Choi JW Increased oxidative metabolism in the Li-Fraumeni syndrome. N Engl J Med (2013) 368:1027–32. 10.1056/NEJMoa1214091 23484829 PMC4123210

[41] Tong X Zhao F Thompson CB The molecular determinants of de novo nucleotide biosynthesis in cancer cells. Curr Opin Genet Dev (2009) 19(1):32–7. 10.1016/j.gde.2009.01.002 19201187 PMC2707261

[42] Mackenzie RW Elliott BT. Akt/PKB activation and insulin signaling: a novel insulin signaling pathway in the treatment of type 2 diabetes. Diabetes Metab Syndr Obes (2014) 7:55–64. 10.2147/DMSO.S48260 24611020 PMC3928478

[43] Matsuda S Kobayashi M Kitagishi Y. Roles for PI3K/AKT/PTEN pathway in cell signaling of nonalcoholic fatty liver disease. ISRN Endocrinol (2013) 2013:472432. 10.1155/2013/472432 23431468 PMC3570922

[44] Izumiya Y Hopkins T Morris C Sato K Zeng L Viereck J Fast/glycolytic muscle fiber growth reduces fat mass and improves metabolic parameters in obese mice. Cell Metab (2008) 7:159–72. 10.1016/J.CMET.2007.11.003 18249175 PMC2828690

[45] Graves LM Guy HI Kozlowski P Huang M Lazarowski E Pope RM Regulation of carbamoyl phosphate synthetase by MAP kinase. Nature (2000) 403:328–32. 10.1038/35002111 10659854

[46] Burotto M Chiou VL Lee J-M Kohn EC. The MAPK pathway across different malignancies: a new perspective. Cancer (2014) 120(22):3446–56. 10.1002/cncr.28864 24948110 PMC4221543

[47] Carlson CJ Koterski S Sciotti RJ Poccard GB Rondinone CM. Enhanced basal activation of mitogen-activated protein kinases in adipocytes from type 2 diabetes: potential role of p38 in the downregulation of GLUT4 expression. Diabetes (2003) 52:634–41. 10.2337/diabetes.52.3.634 12606502

[48] Bost F Aouadi M Caron L Binétruy B. The role of MAPKs in adipocyte differentiation and obesity. Biochimie (2005) 87:51–6. 10.1016/J.BIOCHI.2004.10.018 15733737

[49] Tian WN Braunstein LD Pang J Stuhlmeier KM Xi QC Tian X Importance of glucose-6-phosphate dehydrogenase activity for cell growth. J Biol Chem (1998) 273:10609–17. 10.1074/JBC.273.17.10609 9553122

[50] Patra KC Hay N. The pentose phosphate pathway and cancer. Trends Biochem Sci (2014) 39:347–54. 10.1016/j.tibs.2014.06.005 25037503 PMC4329227

[51] Park J Rho HK Kim KH Choe SS Lee YS Kim JB Overexpression of glucose-6-phosphate dehydrogenase is associated with lipid dysregulation and insulin resistance in obesity. Mol Cell Biol (2005) 25:5146–57. 10.1128/MCB.25.12.5146-5157.2005 15923630 PMC1140588

[52] Carette C Dubois-Laforgue D Gautier J-F Timsit J. Diabetes mellitus and glucose-6-phosphate dehydrogenase deficiency: from one crisis to another. Diabetes Metab (2011) 37:79–82. 10.1016/j.diabet.2010.09.004 21147013

[53] Aird KM Zhang R. Nucleotide metabolism, oncogene-induced senescence and cancer. Cancer Lett (2015) 356:204–10. 10.1016/j.canlet.2014.01.017 24486217 PMC4115046

[54] Engström Y Eriksson S Jildevik I Skog S Thelander L Tribukait B. Cell cycle-dependent expression of mammalian ribonucleotide reductase. Differential regulation of the two subunits. J Biol Chem (1985) 260:9114–6. 3894352

[55] Kolberg M Strand KR Graff P Andersson KK Structure, function, and mechanism of ribonucleotide reductases. Biochim Biophys Acta Proteins Proteomics (2004) 1699(1–2):1–34. 10.1016/j.bbapap.2004.02.007 15158709

[56] Tanaka H Arakawa H Yamaguchi T Shiraishi K Fukuda S Matsui K A ribonucleotide reductase gene involved in a p53-dependent cell-cycle checkpoint for DNA damage. Nature (2000) 404:42–9. 10.1038/35003506 10716435

[57] Bourdon A Minai L Serre V Jais JP Sarzi E Aubert S Mutation of RRM2B, encoding p53-controlled ribonucleotide reductase (p53R2), causes severe mitochondrial DNA depletion. Nat Genet (2007) 39:776–80. 10.1038/ng2040 17486094

[58] Håkansson P Hofer A Thelander L. Regulation of mammalian ribonucleotide reduction and dNTP pools after DNA damage and in resting cells. J Biol Chem (2006) 281:7834–41. 10.1074/jbc.M512894200 16436374

[59] Pontarin G Ferraro P Bee L Reichard P Bianchi V. Mammalian ribonucleotide reductase subunit p53R2 is required for mitochondrial DNA replication and DNA repair in quiescent cells. Proc Natl Acad Sci U S A (2012) 109:13302–7. 10.1073/pnas.1211289109 22847445 PMC3421225

[60] Bornstein B Area E Flanigan KM Ganesh J Jayakar P Swoboda KJ Mitochondrial DNA depletion syndrome due to mutations in the RRM2B gene. Neuromuscul Disord (2008) 18:453–9. 10.1016/j.nmd.2008.04.006 18504129 PMC3891825

[61] Palmieri F Monne M. Discoveries, metabolic roles and diseases of mitochondrial carriers: a review. Biochim Biophys Acta (2016) 1863:2362–78. 10.1016/j.bbamcr.2016.03.007 26968366

[62] Gutierrez-Aguilar M Baines CP. Physiological and pathological roles of mitochondrial SLC25 carriers. Biochem J (2013) 454:371–86. 10.1042/BJ20121753 23988125 PMC3806213

[63] Clemencon B Babot M Trezeguet V. The mitochondrial ADP/ATP carrier (SLC25 family): pathological implications of its dysfunction. Mol Aspects Med (2013) 34:485–93. 10.1016/j.mam.2012.05.006 23506884

[64] Baldwin SA Beal PR Yao SYM King AE Cass CE Young JD. The equilibrative nucleoside transporter family, SLC29. Pflugers Arch (2004) 447:735–43. 10.1007/s00424-003-1103-2 12838422

[65] Gray JH Owen RP Giacomini KM. The concentrative nucleoside transporter family, SLC28. Pflugers Arch (2004) 447:728–34. 10.1007/s00424-003-1107-y 12856181

[66] Molho-Pessach V Lerer I Abeliovich D Agha Z Abu Libdeh A Broshtilova V The H syndrome is caused by mutations in the nucleoside transporter hENT3. Am J Hum Genet (2008) 83:529–34. 10.1016/j.ajhg.2008.09.013 18940313 PMC2561939

[67] Arner ES Eriksson S. Mammalian deoxyribonucleoside kinases. Pharmacol Ther (1995) 67:155–86. 10.1016/0163-7258(95)00015-9 7494863

[68] El-Hattab AW Craigen WJ Scaglia F. Mitochondrial DNA maintenance defects. Biochim Biophys Acta (2017) 1863:1539–55. 10.1016/j.bbadis.2017.02.017 28215579

[69] Freisinger P Fütterer N Lankes E Gempel K Berger TM Spalinger J Hepatocerebral mitochondrial DNA depletion syndrome caused by deoxyguanosine kinase (DGUOK) mutations. Arch Neurol (2006) 63:1129–34. 10.1001/archneur.63.8.1129 16908739

[70] Ronchi D Garone C Bordoni A Gutierrez Rios P Calvo SE Ripolone M Next-generation sequencing reveals DGUOK mutations in adult patients with mitochondrial DNA multiple deletions. Brain (2012) 135:3404–15. 10.1093/brain/aws258 23043144 PMC3501975

[71] Spelbrink JN Li FY Tiranti V Nikali K Yuan QP Tariq M Human mitochondrial DNA deletions associated with mutations in the gene encoding twinkle, a phage T7 gene 4-like protein localized in mitochondria. Nat Genet (2001) 28:223–31. 10.1038/90058 11431692

[72] Copeland WC. Inherited mitochondrial diseases of DNA replication. Annu Rev Med (2008) 59:131–46. 10.1146/annurev.med.59.053006.104646 17892433 PMC2271032

[73] Tyynismaa H Mjosund KP Wanrooij S Lappalainen I Ylikallio E Jalanko A Mutant mitochondrial helicase twinkle causes multiple mtDNA deletions and a late-onset mitochondrial disease in mice. Proc Natl Acad Sci U S A (2005) 102:17687–92. 10.1073/pnas.0505551102 16301523 PMC1308896

[74] Krasich R Copeland WC. DNA polymerases in the mitochondria: a critical review of the evidence. Front Biosci (Landmark Ed) (2017) 22:692–709. 10.2741/4510 27814640 PMC5485829

[75] Singh B Owens KM Bajpai P Desouki MM Srinivasasainagendra V Tiwari HK Mitochondrial DNA polymerase POLG1 disease mutations and germline variants promote tumorigenic properties. PLoS One (2015) 10:e0139846. 10.1371/journal.pone.0139846 26468652 PMC4607296

[76] Singh KK Ayyasamy V Owens KM Koul MS Vujcic M. Mutations in mitochondrial DNA polymerase-gamma promote breast tumorigenesis. J Hum Genet (2009) 54:516–24. 10.1038/jhg.2009.71 19629138 PMC2782392

[77] Tervasmaki A Mantere T Hartikainen JM Kauppila S Lee H-M Koivuluoma S Rare missense mutations in RECQL and POLG associate with inherited predisposition to breast cancer. Int J Cancer (2018). 10.1002/ijc.31259 29341116

[78] Linkowska K Jawien A Marszalek A Malyarchuk BA Tonska K Bartnik E Mitochondrial DNA polymerase gamma mutations and their implications in mtDNA alterations in colorectal cancer. Ann Hum Genet (2015) 79(5):320–8. 10.1111/ahg.12111 25850945

[79] Long X Wang X Chen Y Guo X Zhou F Fan Y Polymorphisms in POLG were associated with the prognosis and mtDNA content in hepatocellular carcinoma patients. Bull Cancer (2017) 104:500–7. 10.1016/j.bulcan.2017.02.005 28457473

[80] Aird KM Worth AJ Snyder NW Lee JV Sivanand S Liu Q ATM couples replication stress and metabolic reprogramming during cellular senescence. Cell Rep (2015) 11:893–901. 10.1016/j.celrep.2015.04.014 25937285 PMC4431925

[81] Choi M Kipps T Kurzrock R. ATM mutations in cancer: therapeutic implications. Mol Cancer Ther (2016) 15:1781–91. 10.1158/1535-7163.MCT-15-0945 27413114

[82] Guleria A Chandna S. ATM kinase: much more than a DNA damage responsive protein. DNA Repair (Amst) (2016) 39:1–20. 10.1016/j.dnarep.2015.12.009 26777338

[83] Parks DA Granger DN Xanthine oxidase: biochemistry, distribution and physiology. Acta Physiol Scand Suppl (1986) 548:87–99. 3529824

[84] Battelli MG Polito L Bortolotti M Bolognesi A Xanthine oxidoreductase in cancer: more than a differentiation marker. Cancer Med (2016) 5:546–57. 10.1002/cam4.601 26687331 PMC4799950

[85] Chen C Lu J-M Yao Q. Hyperuricemia-related diseases and xanthine oxidoreductase (XOR) inhibitors: an overview. Med Sci Monit (2016) 22:2501–12. 10.12659/MSM.899852 27423335 PMC4961276

[86] Kaukonen J Juselius JK Tiranti V Kyttala A Zeviani M Comi GP Role of adenine nucleotide translocator 1 in mtDNA maintenance. Science (2000) 289:782–5. 10.1126/science.289.5480.782 10926541

[87] Favre C Zhdanov A Leahy M Papkovsky D O’Connor R. Mitochondrial pyrimidine nucleotide carrier (PNC1) regulates mitochondrial biogenesis and the invasive phenotype of cancer cells. Oncogene (2010) 29:3964–76. 10.1038/onc.2010.146 20453889

[88] Floyd S Favre C Lasorsa FM Leahy M Trigiante G Stroebel P The insulin-like growth factor-I-mTOR signaling pathway induces the mitochondrial pyrimidine nucleotide carrier to promote cell growth. Mol Biol Cell (2007) 18:3545–55. 10.1091/mbc.E06-12-1109 17596519 PMC1951771

[89] Kenerson HL Subramanian S McIntyre R Kazami M Yeung RS. Livers with constitutive mTORC1 activity resist steatosis independent of feedback suppression of akt. PLoS One (2015) 10(2):e0117000. 10.1371/journal.pone.0117000 25646773 PMC4315590

[90] Um SH Frigerio F Watanabe M Picard F Joaquin M Sticker M Absence of S6K1 protects against age- and diet-induced obesity while enhancing insulin sensitivity. Nature (2004) 431:200–5. 10.1038/nature02866 15306821

[91] Kim LC Cook RS Chen J. MTORC1 and mTORC2 in cancer and the tumor microenvironment. Oncogene (2017) 36(16):2191–201. 10.1038/onc.2016.363 27748764 PMC5393956

[92] Millis SZ Ikeda S Reddy S Gatalica Z Kurzrock R Landscape of phosphatidylinositol-3-kinase pathway alterations across 19 784 diverse solid tumors. JAMA Oncol (2016) 2:1565–73. 10.1001/jamaoncol.2016.0891 27388585

[93] Malley CO Pidgeon GP The mTOR pathway in obesity driven gastrointestinal cancers: potential targets and clinical trials. BBA Clin (2016) 5:29–40. 10.1016/j.bbacli.2015.11.003 27051587 PMC4802403

[94] Sears RC. The life cycle of c-Myc: from synthesis to degradation. Cell Cycle (2004) 3(9):1133–7. 10.4161/cc.3.9.1145 15467447

[95] Miller DM Thomas SD Islam A Muench D Sedoris K c-Myc and cancer metabolism. Clin Cancer Res (2012). 10.1158/1078-0432.CCR-12-0977 PMC3505847 23071356

[96] Wise DR DeBerardinis RJ Mancuso A Sayed N Zhang X-Y Pfeiffer HK Myc regulates a transcriptional program that stimulates mitochondrial glutaminolysis and leads to glutamine addiction. Proc Natl Acad Sci U S A (2008) 105:18782–7. 10.1073/pnas.0810199105 19033189 PMC2596212

[97] Riu E Ferre T Mas A Hidalgo A Franckhauser S Bosch F. Overexpression of c-myc in diabetic mice restores altered expression of the transcription factor genes that regulate liver metabolism. Biochem J (2002) 368:931–7. 10.1042/BJ20020605 12230428 PMC1223040

[98] Yang L Venneti S Nagrath D. Glutaminolysis: a hallmark of cancer metabolism. Annu Rev Biomed Eng (2017) 19:163–94. 10.1146/annurev-bioeng-071516-044546 28301735

[99] DeBerardinis RJ Mancuso A Daikhin E Nissim I Yudkoff M Wehrli S Beyond aerobic glycolysis: transformed cells can engage in glutamine metabolism that exceeds the requirement for protein and nucleotide synthesis. Proc Natl Acad Sci U S A (2007) 104:19345–50. 10.1073/pnas.0709747104 18032601 PMC2148292

[100] Chabes A Georgieva B Domkin V Zhao X Rothstein R Thelander L. Survival of DNA damage in yeast directly depends on increased dNTP levels allowed by relaxed feedback inhibition of ribonucleotide reductase. Cell (2003) 112:391–401. 10.1016/S0092-8674(03)00075-8 12581528

[101] Le TM Poddar S Capri JR Abt ER Kim W Wei L ATR inhibition facilitates targeting of leukemia dependence on convergent nucleotide biosynthetic pathways. Nat Commun (2017) 8:241. 10.1038/s41467-017-00221-3 28808226 PMC5556071

[102] Shimizu I Yoshida Y Katsuno T Tateno K Okada S Moriya J P53-induced adipose tissue inflammation is critically involved in the development of insulin resistance in heart failure. Cell Metab (2012) 15:51–64. 10.1016/j.cmet.2011.12.006 22225876

[103] Jiang P Du W Wu M Regulation of the pentose phosphate pathway in cancer. Protein Cell (2014) 8(8):592–602. 10.1007/s13238-014-0082-8 PMC4112277 25015087

[104] Lee J-W Choi AH Ham M Kim J-W Choe SS Park J G6PD up-regulation promotes pancreatic beta-cell dysfunction. Endocrinology (2011) 152(3):793–803. 10.1210/en.2010-0606 21248143

[105] Ham M Choe SS Shin KC Choi G Kim JW Noh JR Glucose-6-phosphate dehydrogenase deficiency improves insulin resistance with reduced adipose tissue inflammation in obesity. Diabetes (2016) 65:2624–38. 10.2337/db16-0060 27284106

[106] Wang L Pi Z Liu S Liu Z Song F. Targeted metabolome profiling by dual-probe microdialysis sampling and treatment using *Gardenia jasminoides* for rats with type 2 diabetes. Sci Rep (2017) 7:10105. 10.1038/s41598-017-10172-w 28860508 PMC5579158

[107] Xia J-F Liang Q-L Liang X-P Wang Y-M Hu P Li P Ultraviolet and tandem mass spectrometry for simultaneous quantification of 21 pivotal metabolites in plasma from patients with diabetic nephropathy. J Chromatogr B Analyt Technol Biomed Life Sci (2009) 877:1930–6. 10.1016/j.jchromb.2009.05.047 19501555

[108] Li L Liu DW Yan HY Wang ZY Zhao SH Wang B. Obesity is an independent risk factor for non-alcoholic fatty liver disease: evidence from a meta-analysis of 21 cohort studies. Obes Rev (2016) 17:510–9. 10.1111/obr.12407 27020692

[109] Sun B Karin M Obesity, inflammation, and liver cancer. J Hepatol (2012) 56:704–13. 10.1016/j.jhep.2011.09.020 22120206 PMC3889660

[110] Gallagher EJ LeRoith D. Obesity and diabetes: the increased risk of cancer and cancer-related mortality. Physiol Rev (2015) 95:727–48. 10.1152/physrev.00030.2014 26084689 PMC4491542

[111] Dann SG Selvaraj A Thomas G. mTOR complex1-S6K1 signaling: at the crossroads of obesity, diabetes and cancer. Trends Mol Med (2007) 13(6):252–9. 10.1016/j.molmed.2007.04.002 17452018

[112] Aird KM Zhang G Li H Tu Z Bitler BG Garipov A Suppression of nucleotide metabolism underlies the establishment and maintenance of oncogene-induced senescence. Cell Rep (2013) 3:1252–65. 10.1016/j.celrep.2013.03.004 23562156 PMC3840499

[113] Bester AC Roniger M Oren YS Im MM Sarni D Chaoat M Nucleotide deficiency promotes genomic instability in early stages of cancer development. Cell (2011) 145:435–46. 10.1016/j.cell.2011.03.044 21529715 PMC3740329

[114] Fatkhutdinov N Sproesser K Krepler C Liu Q Brafford PA Herlyn M Targeting RRM2 and mutant BRAF is a novel combinatorial strategy for melanoma. Mol Cancer Res (2016) 14:767–75. 10.1158/1541-7786.mcr-16-0099 27297629 PMC5025362

[115] Xu X Page JL Surtees JA Liu H Lagedrost S Lu Y Broad overexpression of ribonucleotide reductase genes in mice specifically induces lung neoplasms. Cancer Res (2008) 68:2652–60. 10.1158/0008-5472.CAN-07-5873 18413732 PMC2459241

[116] Hofer A Crona M Logan DT Sjöberg BM. DNA building blocks: keeping control of manufacture. Crit Rev Biochem Mol Biol (2012) 47(1):50–63. 10.3109/10409238.2011.630372 22050358 PMC3267527

[117] Nordlund P Reichard P. Ribonucleotide reductases. Annu Rev Biochem (2006) 75:681–706. 10.1146/annurev.biochem.75.103004.142443 16756507

[118] Eriksson S Graslund A Skog S Thelander L Tribukait B. Cell cycle-dependent regulation of mammalian ribonucleotide reductase. The S phase-correlated increase in subunit M2 is regulated by de novo protein synthesis. J Biol Chem (1984) 259:11695–700. 6090444

[119] D’Angiolella V Donato V Forrester FM Jeong YT Pellacani C Kudo Y Cyclin F-mediated degradation of ribonucleotide reductase M2 controls genome integrity and DNA repair. Cell (2012) 149:1023–34. 10.1016/j.cell.2012.03.043 22632967 PMC3616325

[120] Foskolou IP Jorgensen C Leszczynska KB Olcina MM Tarhonskaya H Haisma B Ribonucleotide reductase requires subunit switching in hypoxia to maintain DNA replication. Mol Cell (2017) 66:206–220.e9. 10.1016/j.molcel.2017.03.005 28416140 PMC5405111

[121] Kimura T Takeda S Sagiya Y Gotoh M Nakamura Y Arakawa H. Impaired function of p53R2 in Rrm2b-null mice causes severe renal failure through attenuation of dNTP pools. Nat Genet (2003) 34:440–5. 10.1038/ng1212 12858174

[122] Liu X Xue L Yen Y. Redox property of ribonucleotide reductase small subunit M2 and p53R2. Methods Mol Biol (2008) 477:195–206. 10.1007/978-1-60327-517-0_15 19082948

[123] Elledge SJ Zhou Z Allen JB Navas TA. DNA damage and cell cycle regulation of ribonucleotide reductase. Bioessays (1993) 15:333–9. 10.1002/bies.950150507 8343143

[124] Aird KM Li H Xin F Konstantinopoulos PA Zhang RG. Identification of ribonucleotide reductase M2 as a potential target for pro-senescence therapy in epithelial ovarian cancer. Cell Cycle (2014) 13:199–207. 10.4161/cc.26953 24200970 PMC3906237

[125] Dressman HK Hans C Bild A Olson JA Rosen E Marcom PK Gene expression profiles of multiple breast cancer phenotypes and response to neoadjuvant chemotherapy. Clin Cancer Res (2006) 12:819–26. 10.1158/1078-0432.CCR-05-1447 16467094

[126] Fujita H Ohuchida K Mizumoto K Itaba S Ito T Nakata K Gene expression levels as predictive markers of outcome in pancreatic cancer after gemcitabine-based adjuvant chemotherapy. Neoplasia (2010) 12:807–17. 10.1593/neo.10458 20927319 PMC2950330

[127] Liu X Zhou B Xue L Yen F Chu P Un F Ribonucleotide reductase subunits M2 and p53R2 are potential biomarkers for metastasis of colon cancer. Clin Color Cancer (2007) 6:374–81. 10.3816/CCC.2007.n.007 17311703

[128] Sagan L On the origin of mitosing cells. J Theor Biol (1967) 14:225–IN6. 10.1016/0022-5193(67)90079-3 11541392

[129] Galluzzi L Kepp O Trojel-Hansen C Kroemer G. Mitochondrial control of cellular life, stress, and death. Circ Res (2012) 111(9):1198–207. 10.1161/CIRCRESAHA.112.268946 23065343

[130] Gandhi VV Samuels DC. Correlated tissue expression of genes of cytoplasmic and mitochondrial nucleotide metabolisms in normal tissues is disrupted in transformed tissues. Nucleosides Nucleotides Nucleic Acids (2012) 31:112–29. 10.1080/15257770.2011.644101 22303991 PMC3464496

[131] Hirano M DiMauro S Metabolic myopathies. Adv Neurol (2002) 88:217–34. 11908228

[132] Lee W Johnson J Gough DJ Donoghue J Cagnone GLM Vaghjiani V Mitochondrial DNA copy number is regulated by DNA methylation and demethylation of POLGA in stem and cancer cells and their differentiated progeny. Cell Death Dis (2015) 6:e1664. 10.1038/cddis.2015.34 25719248 PMC4669800

[133] Zhou X Kannisto K Curbo S von Dobeln U Hultenby K Isetun S Thymidine kinase 2 deficiency-induced mtDNA depletion in mouse liver leads to defect beta-oxidation. PLoS One (2013) 8:e58843 10.1371/journal.pone.0058843 23505564 PMC3591375

[134] Bao XR Ong S Goldberger O Peng J Sharma R Thompson DA Mitochondrial dysfunction remodels one-carbon metabolism in human cells. Elife (2016) 5:e10575. 10.7554/eLife.10575 27307216 PMC4911214

[135] Nikkanen J Forsström S Euro L Paetau I Kohnz RA Wang L Mitochondrial DNA replication defects disturb cellular dNTP pools and remodel one-carbon metabolism. Cell Metab (2016) 23:635–48. 10.1016/j.cmet.2016.01.019 26924217

[136] Wallace DC Mitochondrial diseases in man and mouse. Science (1999) 283(5407):1482–8. 10.1126/science.283.5407.1482 10066162

[137] Montgomery MK Turner N Mitochondrial dysfunction and insulin resistance: an update. Endocr Connect (2014) 4:R1–15. 10.1530/EC-14-0092 25385852 PMC4261703

[138] Koliaki C Roden M Alterations of mitochondrial function and insulin sensitivity in human obesity and diabetes mellitus. Annu Rev Nutr (2016):1–31. 10.1146/annurev-nutr-071715-050656 27146012

[139] Kamihara J Rana HQ Garber JE. Germline TP53 mutations and the changing landscape of Li-Fraumeni syndrome. Hum Mutat (2014) 35(6):654–62. 10.1002/humu.22559 24706533 PMC13577162

[140] Malkin D. Li-Fraumeni syndrome. Genes Cancer (2011) 2:475–84. 10.1177/1947601911413466 21779515 PMC3135649

[141] Dahl ES Aird KM Ataxia-telangiectasia mutated modulation of carbon metabolism in cancer. Front Oncol (2017) 7:291 10.3389/fonc.2017.00291 29238697 PMC5712564

[142] Bar RS Levis WR Rechler MM Harrison LC Siebert C Podskalny J Extreme insulin resistance in ataxia telangiectasia: defect in affinity of insulin receptors. N Engl J Med (1978) 298:1164–71. 10.1056/NEJM197805252982103 651946

[143] McKinnon PJ. ATM and the molecular pathogenesis of ataxia telangiectasia. Annu Rev Pathol (2012) 7:303–21. 10.1146/annurev-pathol-011811-132509 22035194

[144] McKinnon PJ. ATM and ataxia telangiectasia. EMBO Rep (2004) 5:772–6. 10.1038/sj.embor.7400210 15289825 PMC1299121

[145] Dore MP Davoli A Longo N Marras G Pes GM. Glucose-6-phosphate dehydrogenase deficiency and risk of colorectal cancer in Northern Sardinia: a retrospective observational study. Medicine (Baltimore) (2016) 95:e5254. 10.1097/MD.0000000000005254 27858887 PMC5591135

[146] Manganelli G Masullo U Passarelli S Filosa S. Glucose-6-phosphate dehydrogenase deficiency: disadvantages and possible benefits. Cardiovasc Hematol Disord Drug Targets (2013) 13:73–82. 10.2174/1871529X11313010008 23534950

[147] Pes GM Bassotti G Dore MP. Colorectal cancer mortality in relation to glucose-6-phosphate dehydrogenase deficiency and consanguinity in Sardinia: a spatial correlation analysis. Asian Pac J Cancer Prev (2017) 18:2403–7. 10.22034/APJCP.2017.18.9.2403 28950694 PMC5720643

[148] Fini MA Elias A Johnson RJ Wright RM. Contribution of uric acid to cancer risk, recurrence, and mortality. Clin Transl Med (2012) 1:16. 10.1186/2001-1326-1-16 23369448 PMC3560981

[149] Sautin YY Johnson RJ. Uric acid: the oxidant-antioxidant paradox. Nucleosides Nucleotides Nucleic Acids (2008) 27(6):608–19. 10.1080/15257770802138558 18600514 PMC2895915

[150] Valko M Rhodes CJ Moncol J Izakovic M Mazur M. Free radicals, metals and antioxidants in oxidative stress-induced cancer. Chem Biol Interact (2006) 160(1):1–40. 10.1016/j.cbi.2005.12.009 16430879

[151] Lu W Xu Y Shao X Gao F Li Y Hu J Uric acid produces an inflammatory response through activation of NF-κB in the hypothalamus: implications for the pathogenesis of metabolic disorders. Sci Rep (2015) 5 10.1038/srep12144 PMC4503982 26179594

[152] Reuter S Gupta SC Chaturvedi MM Aggarwal BB. Oxidative stress, inflammation, and cancer: how are they linked? Free Radic Biol Med (2010) 49(11):1603–16. 10.1016/j.freeradbiomed.2010.09.006 20840865 PMC2990475

[153] Galmarini CM Mackey JR Dumontet C. Nucleoside analogues and nucleobases in cancer treatment. Lancet Oncol (2002) 3(7):415–24. 10.1016/S1470-2045(02)00788-X 12142171

[154] Muñoz-Pinedo C El Mjiyad N Ricci J-E. Cancer metabolism: current perspectives and future directions. Cell Death Dis (2012) 3:e248. 10.1038/cddis.2011.123 22237205 PMC3270265

[155] de Sousa Cavalcante L Monteiro G. Gemcitabine: metabolism and molecular mechanisms of action, sensitivity and chemoresistance in pancreatic cancer. Eur J Pharmacol (2014) 741:8–16. 10.1016/j.ejphar.2014.07.041 25084222

[156] Amrutkar M Gladhaug IP Pancreatic cancer chemoresistance to gemcitabine. Cancers (Basel) (2017) 9 10.3390/cancers9110157 PMC5704175 29144412

[157] DeBerardinis RJ Chandel NS Fundamentals of cancer metabolism. Sci Adv (2016) 2(5):e1600200 10.1126/sciadv.1600200 27386546 PMC4928883

